# Single-Cell RNA Sequencing and Its Combination with Protein and DNA Analyses

**DOI:** 10.3390/cells9051130

**Published:** 2020-05-04

**Authors:** Jane Ru Choi, Kar Wey Yong, Jean Yu Choi, Alistair C. Cowie

**Affiliations:** 1 Centre for Blood Research, Life Sciences Centre, University of British Columbia, 2350 Health Sciences Mall, Vancouver, BV V6T 1Z3, Canada; 2 Department of Mechanical Engineering, University of British Columbia, 2054-6250 Applied Science Lane, Vancouver, BC V6T 1Z4, Canada; 3 Department of Surgery, Faculty of Medicine & Dentistry, University of Alberta, Edmonton, AB T6G 2R3, Canada; 4 Ninewells Hospital & Medical School, Faculty of Medicine, University of Dundee, Dow Street, Dundee DD1 5EH, UK; j.y.choi@dundee.ac.uk (J.Y.C.); Acowie001@dundee.ac.uk (A.C.C.)

**Keywords:** single-cell RNA sequencing, protein, genome, biomedical applications, commercialization

## Abstract

Heterogeneity in cell populations poses a significant challenge for understanding complex cell biological processes. The analysis of cells at the single-cell level, especially single-cell RNA sequencing (scRNA-seq), has made it possible to comprehensively dissect cellular heterogeneity and access unobtainable biological information from bulk analysis. Recent efforts have combined scRNA-seq profiles with genomic or proteomic data, and show added value in describing complex cellular heterogeneity than transcriptome measurements alone. With the rising demand for scRNA-seq for biomedical and clinical applications, there is a strong need for a timely and comprehensive review on the scRNA-seq technologies and their potential biomedical applications. In this review, we first discuss the latest state of development by detailing each scRNA-seq technology, including both conventional and microfluidic technologies. We then summarize their advantages and limitations along with their biomedical applications. The efforts of integrating the transcriptome profile with highly multiplexed proteomic and genomic data are thoroughly reviewed with results showing the integrated data being more informative than transcriptome data alone. Lastly, the latest progress toward commercialization, the remaining challenges, and future perspectives on the development of scRNA-seq technologies are briefly discussed.

## 1. Introduction

Single cells are the basic structural, biological, and functional unit of organisms [1,2]. Traditional RNA sequencing of complex tissues usually masks the uniqueness of each cell [3]. Different cells often assume specific roles by collectively contributing to the overall functions of a tissue or organ. Therefore, the transcriptomic analysis at the single-cell level is highly informative in improving understanding of the complexities of biological processes associated with physiological functions and human diseases [4,5]. Single cell RNA-sequencing (scRNA-seq) has revealed the uniqueness of individual cells and, thus, addressed questions unobtainable in bulk analysis [6]. It has been applied to discover new cell types [7,8], explore the dynamics of developmental biological processes [9], and identify gene regulatory mechanisms [10]. For example, through scRNA-seq, different subpopulations of cells can be resolved, which, thereby, enables characterization of a heterogenous cell population [7]. Furthermore, rare cell types can be identified, which provides valuable insights for disease diagnosis and treatment [11]. Therefore, the development of robust scRNA-seq technologies holds a potential in understanding tissue and organ functions at the cellular level, which plays a significant role in contributing to diagnostic and therapeutic medical advancement.

Conventional scRNA-seq technologies initially involved manual isolation of cells using mouth pipettes [12], micropipettes [13,14], or fluorescence activated cell sorting (FACS) [15]. While these technologies can be scaled up and automated, they remain time-consuming and difficult. Recent advances in microfluidic technologies have opened new avenues for scRNA-seq by integrating semi-automated operations into a much simpler device [16,17,18]. For instance, valve-based microfluidic devices have been developed to trap or capture single cells in reaction chambers using microvalves, where the cells are lysed and their mRNAs are reverse transcribed and amplified [19]. Compared to conventional technologies, microfluidic technologies involve fewer operational steps and improved throughput [20,21,22]. Droplet microfluidic devices have also been introduced to encapsulate individual cells in small volume droplets containing reagents [23,24]. Then, cells are lysed and sorted for library preparation and sequencing. Specifically, these technologies offer several advantages: reduced volume of the reagent and sample required, reduced operational steps, high analytical sensitivity and specificity, and high throughput. In addition, Nanowell technologies have been developed to offer several advantages such as ease-of-operation, low sample and reagent volume requirement, and the capability to examine cell phenotypes, such as cell shape and size [25,26]. These capabilities allow users to tune cell loading density, identify doublets or multiplets, determine cell viability, and identify cells of interest for more effective downstream processes.

**Figure 1:**
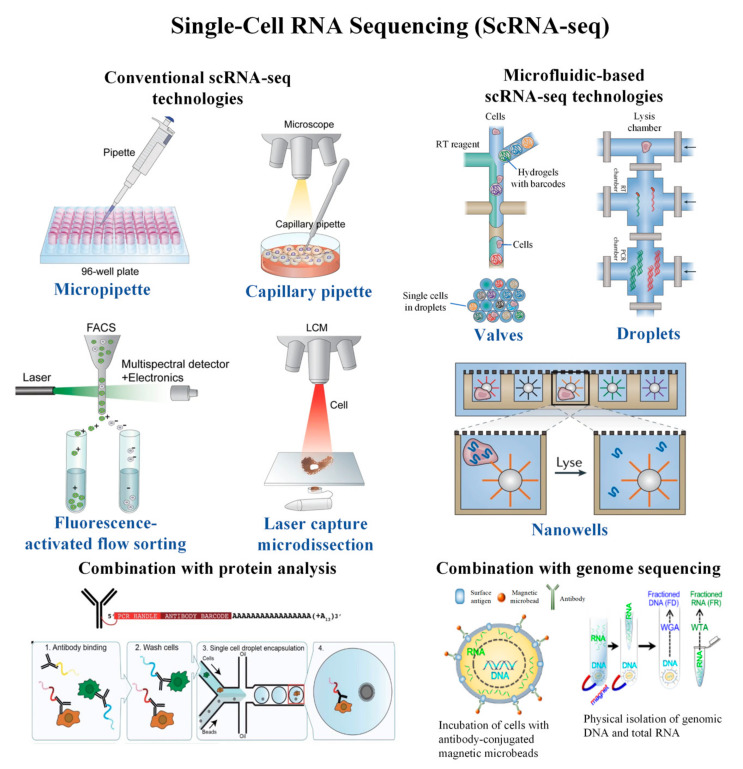
Schematic diagram of single-cell RNA sequencing and its combination with protein and DNA analyses. Conventional scRNA-seq involves isolation of cells using a micropipette, capillary pipette, fluorescence-activated cell sorting, or laser capture microdissection. Microfluidic-based scRNA-seq technologies involve valve-based, droplet-based, and Nanowell-based technologies. The transcriptomic analysis was combined with protein and DNA analyses to provide more informative output from single cells. Adapted with permission from Reference [35] © Creative Commons Attribution License (2018).

Review articles on the introduction of scRNA-seq technologies are readily available [20,21,27,28,29,30]. However, most of them focus primarily on the principle of technologies [31], microfluidic fabrication [32], or multi-omics alone [33,34]. To date, in view of the advancement of the scRNA-seq technologies, there is a strong demand for a timely and comprehensive review on scRNA-seq technologies and their integration with genome and proteomic studies. In the present review, we first discuss the latest development in the field by detailing each scRNA-seq technology, including both conventional and current microfluidic technologies (Figure 1). The combination of scRNA-seq with protein and DNA analysis are comprehensively reviewed. Next, the advantages and limitations of the technologies along with their biomedical applications are highlighted. Lastly, the latest progress toward commercialization, the existing challenges, and future perspectives are discussed.

## 2. Conventional scRNA-seq Technologies

**Figure 2:**
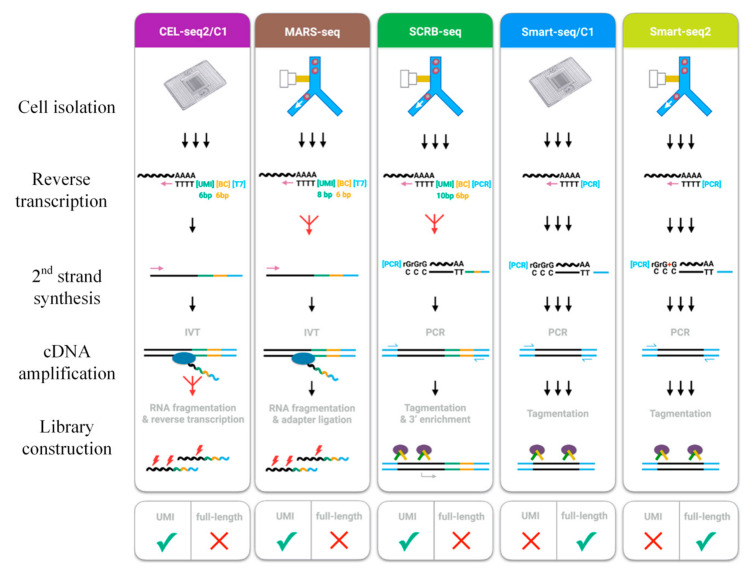
Conventional scRNA-seq. Conventional scRNA-seq technologies include Cel-seq 1/2, MARS-Seq, SCRB-seq, and Smart-seq1/2. The cells are usually isolated using a micropipette, mouth pipette, or fluorescence-activated flow sorting. They are then lysed and undergo reverse transcription and amplification prior to library construction. Adapted with permission from Reference [36] © Elsevier (2017).

Numerous conventional methods exist to isolate single cells for scRNA-seq (Figure 2), and these scRNA-seq technologies are summarized in Table 1. These technologies will be briefly discussed in the following section.

### 2.1. Smart-seq 1 and 2

Switching Mechanism at 5′ End of RNA Template (Smart-seq) has been introduced to address the limitations of existing technologies such as limited throughput and read coverage across transcripts [45]. Briefly, single cells are manually picked and lysed in reverse transcriptase (moloney murine leukemia virus) and the reaction is started with oligo(dT) containing primer. When reverse transcription (RT) reaches the 5′ end of an RNA molecule, a few C nucleotides are added to the 3′ end of the cDNA for the first strand synthesis. In the presence of a template switching oligo (TSO), templates are switched by RT and the second strand of cDNA is synthesized. Full-length cDNAs are subsequently amplified using a polymerase chain reaction (PCR) to obtain a few nanograms of DNA. Illumina sequencing libraries are then prepared according to Nextera Tn5 transposon protocol. This technology dramatically improves transcript coverage and enhances evaluation of single nucleotide polymorphisms or identification of candidate biomarkers. It is particularly useful for investigating the transcriptomic profile in rare cells.

Smart-seq 2 has been introduced to address the challenges of low-yield, coverage, and poor sensitivity in smart-seq 1 [37]. It improves RT, template switching, and preamplification processes to increase length and yield of cDNA libraries generated from single cells. BrieThe last guanylate at the TSO 3′ end is replaced with a locked nucleic acid (LNA) to double the cDNA yield obtained with the TSO in Smart-seq 1 due to the increased thermal stability of LNA-DNA base pairs. The use of methyl group donor betaine and higher concentration of MgCl_2_ significantly improves the cDNA yield. Adding deoxyribonucleoside triphosphates (dNTP) before RNA denaturation increases the pre-amplified cDNA average length possibly due to the stabilization of RNA-oligo(dT) primer hybridization. Utilizing KAPA HiFi Hotstart DNA polymerase during preamplification provides a good amplification efficacy and greater cDNA length (i.e., 450 nt greater). Hence, in addition to stability enhancement, Smart-seq 2 has improved both the length and yield of cDNA libraries generated from single cells as well as the coverage, bias, and accuracy of detection. Time-consuming cell isolation processes using the micropipette and a low number of cells are known as the limitations of Smart-seq.

### 2.2. SCRB-seq

Single Cell RNA Barcoding and Sequencing (SCRB-seq) is introduced to profile mRNAs from a large number of cells using a minimal amount of reagents and sequencing reads per cell [15]. This method, developed according to Smart-seq protocol, only performs 3′ end sequencing with cell specific barcodes and unique molecular identifiers (UMI). Single cells are sorted into a 384-well plate via FACS. RT was carried out using RT primers composed of barcodes, UMI, and poly(T) primer. The resultant cDNA is pooled and amplified for sequencing using a fragmentation approach that enriches 3′ ends. SCRB-seq allows deep, full-length transcriptome coverage sequencing and is able to sequence about 12,000 single cells. Unlike Smart-seq, this technology includes the use of cell barcodes to enable easier identification of reads that originate from the same cell. However, sequencing larger numbers of single cells remains challenging. This method is suitable for discovering transcriptomes across heterogeneous populations.

### 2.3. CEL-seq 1 and 2

Cell Expression by Linear Amplification and Sequencing (CEL-seq) mainly relies on linear amplification of CDNA by in vitro transcription. This protocol allows pooling of barcoded samples. Therefore, this dramatically improves the amplification efficiency [13]. Single cells are manually transferred into tubes using micropipettes. After each cell is lysed, a tailed oligo(dT) is used to prime RT. From the 5′ end to the 3′ end, the sequence of the tailed oligo(dT) is a T7 promoter, partial Illumina 5′ adapter, cell barcode, and poly(T) primer. The second-strand cDNA is then synthesized to generate a double-stranded cDNA containing a T7 promoter. The cDNAs are pooled and an in vitro transcription reaction is initiated to achieve linear amplification of cDNA. The amplicons generated are fragmented to a size distribution suitable for sequencing. This technology was applied to study sister cells from early *C. elegans* embryos and demonstrated the possibility of distinguishing cell types even in the presence of only subtle biological differences.

Essentially, CEL-seq, which involves 3′ end cDNA coverage, gives a more sensitive and reproducible outcome than full length cDNA coverage. Compared to Smart-seq, CEL-seq adds the barcode at an earlier stage, which specifically identifies each single cell. Hence, this reduces the hands-on work. However, this technology can only be used for 3′-end sequencing, which provides less transcriptomic information than full length transcript sequencing.

CEL-seq 2, which is a modified method of CEL-Seq, adds a 5-base pair UMI upstream of the barcode to identify PCR duplicates in scRNA-seq [14], which significantly improves the accuracy. The utilization of the Super-Script II Double-Stranded cDNA Synthesis Kit in combination with a shortening of the CEL-seq primer dramatically improves RT efficiency, which, thereby, increases the detection sensitivity. In addition, 30% more genes are able to be detected by CEL-seq 2 as compared to the original CEL-seq protocol. Off-the-shelf reagents are also used to generate single-cell transcriptome libraries, which makes them accessible to most laboratories. In contrast to Smart-seq, the use of cell barcodes in CEL-seq enables better identification of single cells. Similar to Smart-seq, CEL-seq uses a micropipette for cell isolation, which makes the processes time-consuming.

### 2.4. MARS-seq 1 and 2

Massively Parallel RNA Single-Cell Sequencing (MARS-seq) was introduced following a CEL-seq protocol as an automated workflow to analyze transcriptomes of thousands of single cells while minimizing amplification biases and labeling errors [39]. Single cells are sorted into 384 well plates through FACS and RT is performed with a T7 promoter, a partial Illumina adapter, a cell barcode, a UMI, and a poly(T) primer. Subsequently, automated processing is performed on pooled and labeled materials with three levels of barcoding (molecular, cellular, and plate level), which dramatically increases throughput and reproducibility. It could be applied to define cell type and cell state and link these to detailed genome wide transcriptomic profiling.

MARS-seq 2 is a modified method of MARS-seq that incorporates indexed FACS sorting to enrich cells of interest. This key feature is vital for identification of rare cell subpopulations via scRNA-seq [40], such as a unique microglia that restrict the development of Alzheimer’s disease [46]. Compared to MARS-seq, experimental improvements, such as optimization of RT primer concentration and composition and addition of RT primer removal step in MARS-seq 2, greatly reduce technical cell-to-cell contamination (background noise). Additionally, MARS-seq 2 minimizes cell doublets per well (0.2%) that complicate the scRNA-seq analysis. This technology performs FACS requiring skilled workers. However, due to its automated processes, it minimizes sampling bias and simplifies user steps compared to the above-mentioned technologies.

### 2.5. Quartz-seq 1 and 2

Quartz-seq is a simple and highly-quantitative scRNA-seq approach based on homopolymer tailing-based PCR [41]. Besides assessing transcriptome heterogeneity between the same type of cells, it also detects transcriptome heterogeneity between the cells in the same cell-cycle phase. Since homopolymer tailing-based PCR tends to generate unexpected byproducts that complicate the scRNA-seq analysis, Quartz-seq adds an RT primer removal step and uses suppression PCR technology to reduce synthesis of byproducts. This eliminates the need for complicated byproduct removal methods. Single cells are sorted into tubes through FACS and lysed. mRNA is reverse transcribed to first-strand cDNA using RT primer that contains a PCR target region. Unreacted RT primer is digested by exonuclease 1 and a poly(A) tail is added to the 3′ ends of the cDNA and to any remaining RT primer. The second-strand cDNA synthesis is performed using a tagging primer that contains a poly (dT) sequence, which results in both cDNA and byproducts that contain whole transcriptome amplification (WTA) adaptor sequences (tagging and RT primer sequences). These DNAs are then subjected to suppression PCR to remove the byproducts and obtain high-quality cDNA for Illumina sequencing. Quartz-seq is simply compared to the Kurimoto et al. method [47] that requires multiple PCR tubes for a single cell. Compared to Smart-seq, Quartz-seq is highly quantitative, which detects more transcripts.

A key feature of Quartz-seq 2 is its high capability of analyzing single cell transcriptome with a limited number of sequence reads [42]. Single cells are sorted into a 384-well plate through FACS and a cell barcoding strategy is performed using RT primer that contains a UMI sequence and a cell barcode sequence. Additionally, the efficiency of poly(A) tail tagging strategy is improved by optimization of buffer for the poly(A) tailing step and addition of the increment temperature condition for the second-strand cDNA synthesis step. The UMI conversion efficiency of Quartz-seq 2 (32%–35%) was higher than those of CEL-seq 2, SCRB-seq, and MARS-seq (7%–22%), which allows Quartz-seq 2 to detect more transcripts from limited sequence reads at a minimal cost. Similar to MARS-seq, this technology involves FACS that requires skilled workers.

### 2.6. SUPeR-seq

Single-cell universal poly(A)-independent RNA sequencing (SUPeR-seq) is a scRNA-seq approach based on homopolymer tailing-based PCR that can sequence both polyadenylated and non-polyadenylated RNAs [43]. Circular RNA, which is a non-polyadenylated RNA, is formed by alternate splicing, and is thought to bind and repress several important cellular functions in microRNA [48,49]. Single cells are manually picked up by mouth pipette and lysed to release polyadenylated and non-polyadenylated RNAs. Both RNAs are then subjected to first-strand cDNA synthesis using random primers with a fixed anchor sequence (AnchorX-T_15_N_6_). With the use of ExoSAP-IT, the unreacted primers are removed. The poly(A) tail is added to the 3′ end of the first-stand cDNA using dATP doped with 1% ddATP. Poly(T) primers with a different anchor sequence (AnchorY-T_24_) are then used to synthesize second-strand cDNA, which is followed by PCR using AnchorY-T_24_ and AnchorX-T_15_ primers prior to deep sequencing. This technology was used to study the roles of circular RNAs in mammalian early embryonic development and demonstrated its capability of detecting both circular and polyadenylated RNAs in the mouse preimplantation embryo [43]. Time-consuming cell isolation processes using the mouth pipette and low throughput are known as the drawbacks of SUPeR-seq.

### 2.7. MATQ-seq

Unlike SUPeR-seq, multiple annealing and dC-tailing-based quantitative single-cell RNA-seq (MATQ-seq) incorporates UMI and barcode to sequence both polyadenylated and non-polyadenylated RNAs [44]. Each single cell is mouth pipetted into a PCR tube and lysed to release total RNA. Non-polyadenylated RNA and polyadenylated RNA are subjected to first-strand cDNA synthesis using primers based on multiple annealing and looping-based amplification cycles (MALBAC) that mainly contain T, A, and G bases and MALBAC-dT primers, respectively. The first-stand cDNA is then subjected to poly(C) tailing, which is followed by second-strand cDNA synthesis using G-enriched MALBAC primers. Random hexamer UMI sequences are introduced to label the second-strand cDNA before PCR amplification for Illumina sequencing. MATQ-seq demonstrated that the use of UMI significantly reduces 3′- or 5′-end bias in HEK293T transcripts compared to Smart-seq 2 and SUPeR-seq. MATQ-seq was found to be more sensitive than Smart-seq 2 and SUPeR-seq in detecting non-polyadenylated RNA extracted from single HEK293T cells. Additionally, the capability of detecting low abundance genes using MATQ-seq was higher than that of Smart-seq2. Overall, MATQ-seq provides high accuracy and sensitivity for detecting transcriptomic heterogeneity between single cells of a similar cell type. Similar to SUPeR-seq, MATQ-seq uses mouth pipette for cell isolation, which makes the processes time-consuming and low throughput.

## 3. Microfluidic-Based scRNA-seq Technologies

While the previously mentioned technologies can be automated and scaled to reduce assay costs and reaction volumes, they remain labor-intensive and time-consuming. To this end, several microfluidic technologies have been developed, such as valve-based, droplet-based, and Nanowell-based scRNA-seq technologies. Microfluidic-based scRNA-seq technologies are summarized in Table 2. These technologies allow the sequencing of thousands of cells in a cost-effective manner, which are explicitly discussed below.

### 3.1. Valve-Based scRNA-seq Technologies

One technology to isolate single cells for downstream scRNA-seq is valve-based technology. Valve-based technologies usually rely on dedicated structures for operation such as channels and a pressure controller. They typically perform better than conventional scRNA-seq technologies since they achieve higher reproducibility due to reduction in variation caused by manual handling and pipetting, which leads to a higher sensitivity and accuracy, higher throughput, and lower risk of cross-contamination.

#### 3.1.1. Multilayer Microfluidic Device for scRNA-seq

**Figure 3:**
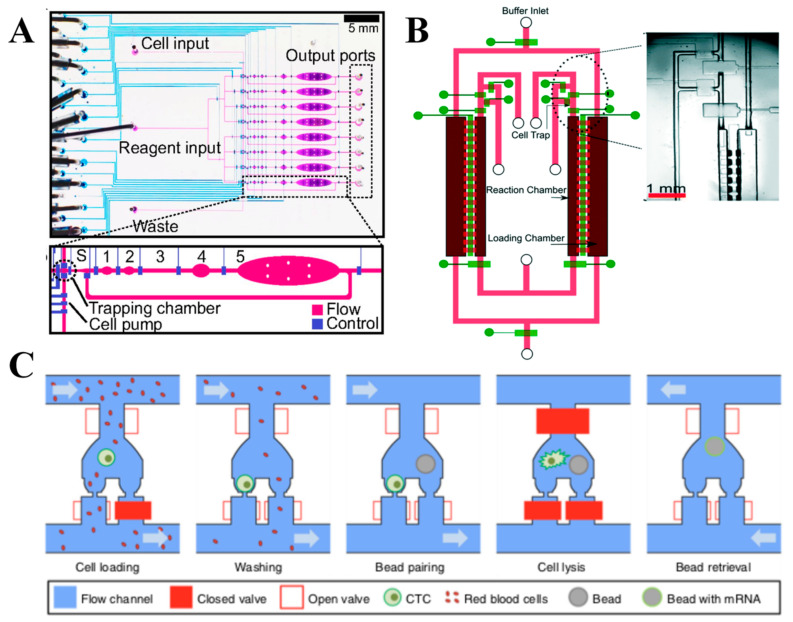
Valve-based scRNA-seq technologies. (**A**) A multilayer microfluidic device with integrated microvalves was developed to prepare cDNA from single cells for scRNA-seq with improved sensitivity and precision. Adapted with permission from Reference [19] © Creative Commons Attribution License (2014). (**B**) MID-RNA-seq technology consists of cell trap, buffer inlet, loading, and reaction chambers to trap and isolate single cells with a diffusion-based reagent swapping scheme, which enables automation and multiplexing. Adapted with permission from Reference [51] © The Royal Society of Chemistry (2019). (**C**) Workflow of Hydro-seq that utilizes a sized-based single cell capture scheme to trap rare cells, such as circulating tumor cells (CTCs), while achieving >70% cell capture efficiency for downstream scRNA-seq. Adapted with permission from Reference [52] © Creative Commons Attribution License (2019).

To overcome the drawbacks of the conventional technologies, a multilayer microfluidic device with integrated valves was developed to prepare cDNA from single cells for scRNA-seq with improved sensitivity and precision (Figure 3A) [19]. A single-cell suspension is obtained from cultured cells and injected into the inlet channel. The single cells are trapped and sorted, and subsequent reactions for cell lysis and RT are conducted, which is followed by off-chip amplification and library preparation. The semi-automated procedure allows a consistent operation time (e.g., loading and mixing), which minimizes technical errors and improves reproducibility. The total reaction volume of all steps is about 140 μL, which is approximately 600-fold lower than the conventional benchtop technology (90 μL). However, the drawbacks of this technology are the requirement of off-chip amplification and low throughput. This technology can identify differentially expressed genes of single cells, which present a great promise to measure biological variations in cell populations in a sensitive and precise manner.

#### 3.1.2. Microfluidic Hydrodynamic Trap Array for scRNA-seq

A microfluidic hydrodynamic trap array was developed to enable off-chip transcriptomic sequencing of single cells after multi-generational lineage tracking under the control of culture conditions [50]. The array consists of 20 lanes of traps. The independent control of pressures with valves enables continuous perfusion for long-term growth and release of single cells from the device. Single cells are loaded into each lane of the trap array and incubated up to 72 h for cell proliferation. Once the cell proliferates, progeny is delivered and captured in a subsequent trap. The entire process undergoes time-lapse imaging, which allows the determination of proliferation kinetics and lineage tracking. The cells are sequenced following the Smart-seq 2 protocol that links the transcriptional measurements to lineage information. Grow kinetics in the device are stable for a long-term culture with consistent doubling time, which demonstrates that the device does not perturb long-term cell proliferation [50]. This technology offers the potential to study multigenerational development at single-cell resolution, which is important for the fields of cancer, immunology, and developmental biology.

#### 3.1.3. MID-RNA-seq

Microfluidic diffusion-based RNA-seq (MID-RNA-seq) was introduced for performing scRNA-seq with a diffusion-based reagent swapping scheme (Figure 3B) [51]. The device incorporates cell trapping, lysis, RT, and PCR amplification with the fluid flow controlled by pneumatic microvalves. This technology leverages an advantage of concentration-gradient-driven diffusion to transport reagents into a reaction chamber while eliminating reagents from the previous steps.

The single-cell suspension was introduced into the device via the sample inlet. Through operating valves, single cells were trapped in chambers. The chambers were rinsed with phosphate buffered saline (PBS) and then with lysis buffer. The lysis buffer was diffused, which moved the trapped cells into the reaction chamber for the lysis reaction. RT and PCR were performed based on the similar diffusion processes, and the chambers were washed with elution buffer to collect cDNA for library preparation and sequencing. However, unlike most conventional technologies, it enables automated processing and multiplexing. The result obtained by this technology was comparable to that of the conventional scRNA-seq technologies. Like the above-mentioned valve-based microfluidic technologies, this technology requires complex device fabrication processes.

#### 3.1.4. Hydro-seq

Hydrodynamic scRNA-seq (Hydro-seq) was introduced to address the challenges of low-throughput and poor cell capture efficiency exhibited by the existing scRNA-seq technologies (Figure 3C) [52]. Hydro-seq utilizes a size-based single cell capture scheme to trap rare cells, such as circulating tumor cells (CTCs), which achieves >70% cell capture efficiency. Once the sample is loaded, the capture valve is closed, and the cells flow through the capture sites where the target cells are trapped in the chamber along with a bead. Lysis buffer is injected and all valves within the chamber are closed. The cell is lysed and its mRNAs are captured by the bead. The bead is then retrieved by opening all valves and introducing a back flow for downstream scRNA-seq. The chamber can be scaled up to thousands for massively parallel enrichment and analysis of rare cells. Compared to other technologies, this technology improved cell capture efficiency and throughput. The utility of hydro-seq was demonstrated by sequencing CTCs and by identifying transcriptome heterogeneity in tumor biomarkers in order to understand metastatic processes and monitor target therapeutics in cancer patients.

### 3.2. Droplet-Based scRNA-seq Technologies

Droplet microfluidic technologies were introduced and typically involve the steps of encapsulating single cells in droplets in an inert carrier oil. The use of carrier oil allows the droplets to be moved, merged, split, heated, or stored. These technologies are fast and have high-throughput. Additionally, compartmentalization of up to thousands of cells can be performed in seconds, which is ideal for large-scale applications.

#### 3.2.1. Hi-SCL

The first droplet-based microfluidic technology developed for scRNA-seq was High-Throughput Single-Cell Labeling (Hi-SCL) [53]. This technology encapsulates single cells in droplets that significantly reduces the volume in which each cell in enclosed, particularly in comparison to the microliter volume commonly used with traditional Nanowell-based technologies. Each droplet is then fused with another droplets containing lysis buffer, RT buffer, and DNA barcodes that uniquely label the single-cell transcriptome. The droplets are subsequently amplified and sequenced. The low risk of contamination between droplets in emulsion was demonstrated by mixing human and mouse cells, which showed most reads were obtained from each unique species. This technology was applied to measure the RNA levels of hundreds of cells from both mouse embryonic fibroblasts and mouse embryonic stem cells. Essentially, even though this technology has low cell capture efficiency, it enables barcoding and increases capacity of screening a larger quantity of cells. This shows a potential to dramatically extend the capability of microfluidics to characterize single cells in a high throughput manner.

#### 3.2.2. In-Drop

**Figure 4:**
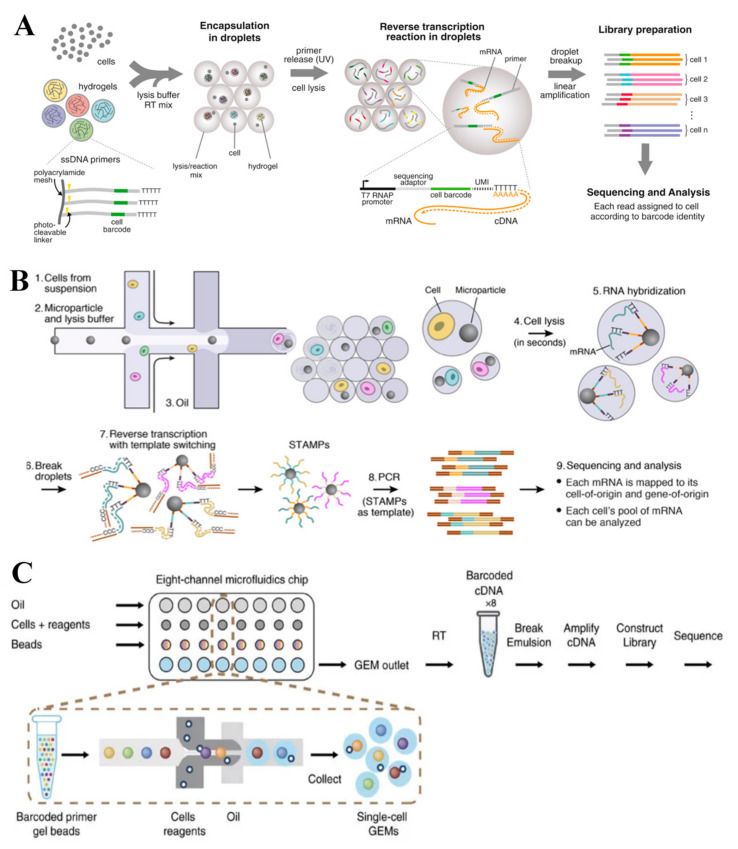
Droplet-based scRNA-seq technologies. (**A**) In-Drop uses hydrogel microspheres to introduce oligonucleotides and all reactions are carried out in droplets, which allows the indexing of thousands of cells for RNA-seq. Adapted with permission from Reference [24] © Elsevier (2015). (**B**) Drop-seq includes a droplet that encapsulates each single cell with a barcode, which enables fast, cost-effective, and high-throughput single-cell analysis. Adapted with permission from Reference [23] © Elsevier (2015). (**C**) 10x genomics uses Gel bead in Emulsion (GEM) to introduce oligonucleotides, and both cell lysis and reverse transcription are introduced in droplets. The use of 10× barcodes significantly increases throughput. Adapted with permission from Reference [54] © Creative Commons Attribution License (2017).

Similar to Hi-SCL, reactions of indexing droplets (In-Drop) are carried out in droplets, which allows the indexing of thousands of cells for RNA-seq (Figure 4A) [24]. The microfluidic device was developed to consist of four inlets for introducing carrier oil, cells, lysis, or RT reagents, a hydrogel microsphere carrying barcoded primers, and one outlet for droplet collection. The hydrogel microspheres are covalently linked to the cell barcodes via a photo-releasable bond. Each barcode consists of a cell barcode, a UMI, an Illumina sequencing primer, a promoter for the T7 RNA polymerase, and an oligo(dT) tail. After cell encapsulation, the cells are lysed and the barcodes are released from the microspheres by exposing the solution to UV light. cDNA in each droplet is then tagged with a barcode during RT, and are amplified and sequenced according to the CEL-seq protocol. The method was optimized to minimize the risk of encapsulating doublets, and yielding >90% droplets containing one cell and one microsphere. This technology enables the scRNA-seq of large numbers of cells, which allows the identification of very rare cell types from heterogeneous populations. Compared to the above-mentioned technologies, In-Drop uses hydrogel microspheres to introduce the oligonucleotides. Lysis and RT are done in droplets to simplify the operation processes. However, the major drawback of In-Drop is the extremely low cell capture efficiency (~7%), which could only detect transcripts present at 20–50 copies per cell.

#### 3.2.3. Drop-seq

Drop-seq shares some similarities with In-Drop, which also involves a droplet that encapsulates each single cell with a barcode (Figure 4B) [23]. Unlike In-Drop that used barcoded hydrogel microspheres, this technology utilizes barcoded beads. The oligonucleotide on beads consist of a handle sequence for amplification, a cell barcode that identifies all oligonucleotides from a single cell, a UMI, and an oligo(dT) sequence that captures single-cell mRNA molecules.

The cells are lysed after being isolated in droplets, and the poly(A) tail of mRNA molecules are hybridized to the oligo(dT) tail on the beads, which forms Single-cell Transcriptomes Attached to MicroParticles (STAMPs). The droplets are then broken and subjected to RT in a single tube. PCR and fragmentation are subsequently performed using the Nextera XT kit. This technology enables high throughput analysis (~10,000 single cell libraries per day) in a cheaper and faster way when compared to the previously mentioned technologies. The cell capture efficiency is ~12.8%, which is higher than that of In-Drop. However, similar to In-Drop, only the 3′ most terminal fragments can be used for sequencing.

#### 3.2.4. 10x Genomics

As mentioned, droplet-based technologies have enabled rapid processing thousands of cells simultaneously, but current technologies has medium throughput, which requires the utilization of custom reagents (Figure 4C) [54]. To overcome these drawbacks, a droplet-based technology, namely 10x Genomics, was developed to enable digital counting of 3′ messenger RNA (mRNA) from thousands of single cells.

Gel bead in Emulsion (GEM) is the core of this technology that is integrated into a droplet-based microfluidic device that efficiently captures approximately 50% of cells loaded. Each gel bead is functionalized with a barcoded oligonucleotide that consists of illumina adapters, 10x barcodes, UMI, and oligo(dT) that prime RT of polyadenylated RNAs. Cell lysis starts immediately after cell encapsulation. Then gel beads dissolve and release the oligonucleotides for RT. RT is performed inside droplets and transferred to a tube where amplification of cDNAs occurs. The amplicons that have Illumina adapters and sample indices allow pooling and sequencing of multiple libraries simultaneously. The sequencing data can be rapidly processed using an analysis pipeline. This technology was utilized to profile 68k peripheral blood mononuclear cells (PBMCs), which demonstrates its ability to dissect large immune populations [54]. Similar to In-Drop, 10x genomics uses gel beads to introduce the oligonucleotides, and both lysis and RT are performed in droplets. It has greatly simplified the entire cell lysis-to-PCR processing time (<10 h). Compared to the existing droplet-based technologies, the introduction of 10x barcodes by this technology has significantly increased throughput. This enabled parallel processing of thousands of cells for scRNA-seq. Similar to In-drop and Drop-seq, only the 3′ most terminal fragments can be used for sequencing.

#### 3.2.5. MULTI-seq

Multiplexing using lipid-tagged indices for single-cell RNA sequencing (MULTI-seq) allows localization of the DNA barcode to a single cell within an emulsion droplet for scRNA-seq [55]. Single cells are first labeled by hybridization of a pair of lipid-modified oligonucleotides (LMO) incorporating 5′ lignoceric acid amide and 3′ palmitic acid amide, respectively, to the cell plasma membranes. The LMO include a 5′ PCR handle, an 8-bp DNA barcode, and a 3′ poly(A) capture sequence. Each single cell carries the LMOs encapsulated together with an mRNA capture bead into an emulsion droplet using the 10x Genomics Chromium system, which is followed by cell lysis to release RNA and LMOs. Both cell mRNA and LMOs hybridize to the mRNA capture bead and then link to a common cell barcode during RT, which allows sample demultiplexing. Following amplification, the LMO fragments are separated from the mRNA by size selection before next generation sequencing (NGS) library preparation. This technology was used to multiplex cryopreserved lungs and primary breast tumors dissected from patient-derived xenograft mouse models at different metastatic progression stages [55]. The utility of MULTI-seq was successfully demonstrated by revealing several immune cell responses toward metastasis of breast tumors to lungs. Since each single cell is labeled with multiple indices, MULTI-seq is able to readily identify and remove cell doublets and improve the throughput of the device. A drawback of this technology is that only the 3′ most terminal fragments can be used for sequencing.

### 3.3. Nanowell-Based scRNA-seq Technologies

More recently, Nanowell technologies were developed, which offer several benefits over droplet-based devices for single cell analysis including a short cell-loading period, low reagent and sample volumes, and enhanced compatibility with optical imaging [57]. The capability of performing optical imaging allows users to examine and tune cell loading density, identify multiplets, and determine cell viability by providing more cellular information prior to library preparation. These technologies will be briefly discussed in the following sections.

#### 3.3.1. Cytoseq

Gene expression cytometry (Cytoseq) enables massively parallel, stochastic barcoding of RNA from single cells in bead-containing Nanowells, which allows for simultaneous gene expression profiling of thousands of single cells [56]. Cells are added into Nanowells and confirmed by microscopy. The beads are loaded to saturate all wells and fresh lysis buffer are added. By placing a magnet on the Nanowell array, the beads with captured mRNAs are retrieved. cDNA synthesis is subsequently carried out using Superscript II or III before performing amplification and sequencing. Similar to Drop-seq, all mRNA molecules in a cell is labelled with a unique cellular barcode and each transcript is indexed with a UMI, which enables mRNA transcripts to be digitally counted. The utility of Cytoseq was demonstrated by identifying rare cells and characterizing cellular heterogeneity in the immune response [56]. Even though this technology is not fully automated, it enables simple fabrication processes and high throughput analysis. This technology will help better understand cellular diversity in complex biological systems for future clinical applications.

#### 3.3.2. Microwell-seq

**Figure 5:**
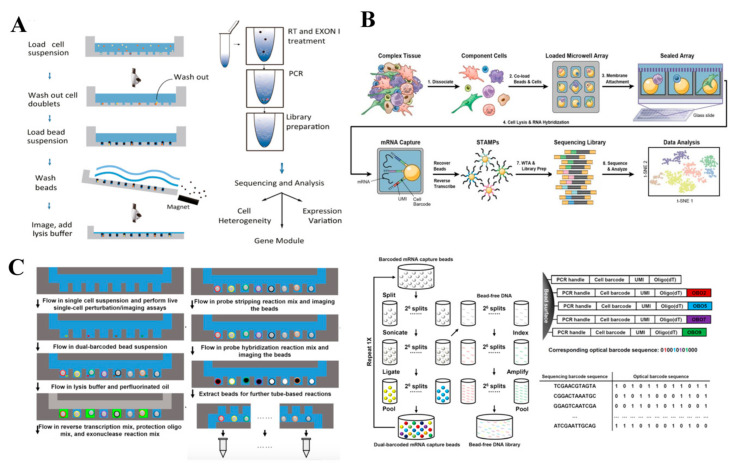
Nanowell-based scRNA-seq technologies. (**A**) Microwell-seq utilizes agarose-constructed Nanowells to profile thousands of single cells. Adapted with permission from Reference [26] © Elsevier, Netherlands(2018). (**B**) Seq-well leverages an advantage of arrays of Nanowells with the use of a semipermeable membrane to reduce cross contamination between wells in order to achieve massively parallel scRNA-seq. Adapted with permission from Reference [25] © Springer Nature (2017). (**C**) (i) Workflow of SCOPE-seq, a technology which is able to identify each individual cell based on their phenotypic profile and link phenotypic information to scRNA-seq data using dual-barcoded mRNA capture beads. (ii) Synthesis process of dual-barcoded mRNA capture beads. Adapted with permission from Reference [57] © Creative Commons Attribution License (2018).

To perform more comprehensive transcriptomic analysis of cell populations, microwell-seq utilized agarose-constructed Nanowells to profile thousands of single cells (Figure 5A) [26]. The silicon microarray was used to construct a micropillar polydimethylsiloxane (PDMS) chip, which was subsequently used to create agarose Nanowell arrays. Like Cytoseq, each magnetic bead is loaded into wells and retrieved by a magnet after capturing single cell mRNAs. RT, amplification, and library preparation are performed following the Smart-seq 2 protocol. Using this technology, a first stage “mouse cell atlas” was constructed with over 400k single-cell transcriptomic profiles from 51 mouse tissues, organs, and cells, which covers more than 800 major cell types and 1000 cell subtypes in the mouse system [26]. Unlike Cytoseq, this technology is able to remove cell doublets based on imaging prior to sample processing for scRNA-seq, which produces a higher quality data. Like other Nanowell technologies, this technology is not fully automated. Future work should further simplify an operational process and integrate data to create a comprehensive mammalian cell map that would be helpful in scientific research and clinical applications.

#### 3.3.3. Seq-Well

More recently, Seq-well was developed, which leveraged the advantage of Nanowell arrays to achieve massively parallel scRNA-seq (Figure 5B) [25]. The thin layer of PDMS Nanowells were fabricated on a glass slide. Cell lysis and RT were conducted on-chip. A key advantage of Seq-well is the use of a semipermeable polycarbonate membrane (10-nm pore size) that is reversibly attached to Nanowells through selective chemical functionalization. This feature allows fast solution exchange to lyse single cells and trap biological macromolecules to minimize cross-contamination, which achieves highly-efficient capture of mRNAs.

The array’s three-layer functionalized surface consists of an amino-silane base crosslinked to a bifunctional poly(glutamate)–chitosan through a p-phenylene diisothiocyanate intermediate. Chitosan on the array’s top surface allows efficient sealing to the membrane while poly(glutamate) on the array’s inner surface inhibits nonspecific mRNA binding. Following cell lysis, the arrays are transferred to new dishes and inverted so that the PDMS surfaces is in contact with the dishes to allow beads to be collected after centrifugation. The library preparation is performed based on a Drop-seq protocol. This technology was used to sequence thousands of primary human macrophages exposed to *Mycobacterium tuberculosis*. It is able to process small amounts of samples and is compatible with on-array imaging cytometry for identifying cell phenotypes from complex biological samples.

#### 3.3.4. SCOPE-seq

Single cell optical phenotyping and expression sequencing (SCOPE-seq) was developed, which enables identification of each individual cell based on their phenotypic profile and link phenotypic information to scRNA-seq data (Figure 5C) [57]. Optically decodable or dual barcoded beads are used, which are generated from Drop-seq beads by attaching unique combinations of oligonucleotides selected from a set of 12 in two cycles of split-pool ligation. Using an optical barcode, the sample identity was linked to a measurement, allowing each optically decodable bead to be decoded by sequential fluorescence hybridization. The beads are conjugated to oligonucleotides that consist of a cell barcode, UMI, and a 3′-poly(dT) sequence used for library preparation and RNA sequencing. The beads and cells are randomly distributed in the PDMS Nanowell array and the array is then cut into multiple pieces once the single cell mRNA is captured by the beads. The beads are then extracted for library preparation. Lastly, the images are processed to identify the optical barcode on each bead and recognize the corresponding barcode to link single cell microscopy data such as cell viability, multiplet detection, cell/nuclei size and morphology, surface marker protein expression level, cell signaling dynamics, and behavior to the RNA-seq data. Like Seq-well, this technology has included the sealing of Nanowells with perfluorinated oil reducing well-to-well contamination, and it is not fully automated. In the future, the beads can be prepared on a large scale, which makes it a powerful technology for associating high throughput microscopic images with sequencing data.

#### 3.3.5. scFTD-seq

To further simplify user steps, single-cell freeze-thaw lysis directly toward 3′ mRNA sequencing (scFTD-seq) of a microchip was developed to perform scRNA-seq using cells that have undergone freeze-thaw lysis [58]. Similar to the previously mentioned technologies, this technology utilized a PDMS Nanowell array. The cells and beads are loaded to the Nanowell arrays, and weak lysis buffer is introduced. For closed Nanowells, the glass slide is then used to carefully seal the array while, for open Nanowells, fluorinated oil is used as a sealant to reduce cross-contamination. The freeze-thaw lysis method is applied to cells in each bead-containing Nanowell and capture mRNAs for transcriptomic sequencing. Similar to the Seq-well approach, the array is transferred to a well plate filled with PBS in an inverted orientation, which is centrifuged to release the beads into the PBS solution. The solution is then transferred to a centrifuge tube to proceed with a downstream RT process. The freeze-thaw lysis described eliminates the requirement for automated fluid exchange that typically requires a complicated microfluidic device. Since there is no active lysing component in the freeze-thaw lysis buffer, this technology does not initiate lysis immediately, which minimizes cross-contamination. It is compatible with both open-environment or close-environment cell loading configurations, which makes it highly suitable to be applied at both the point-of-care setting and centralized laboratories.

## 4. Combination of scRNA-seq with Proteomic Analysis

As previously mentioned, the high throughput scRNA-seq approaches have proven to be valuable for describing complex cell populations. However, the existing approaches do not provide proteomic information such as expression of cell surface proteins. Paired RNA and protein analyses reveal the information of genetic expression and the cell phenotype by providing a more detailed cell subpopulation classification [59]. Some special bioinformatic software (e.g., Cite-seq count) have been introduced to count UMI or antibodies-tagged oligonucleotides in raw sequencing reads. To measure both the transcriptome and cellular proteins in parallel and produce an efficient readout from single cells, several microfluidic technologies have been developed. These technologies are summarized in Table 3 and are described in the following sections.

### 4.1. Cite-seq

**Figure 6:**
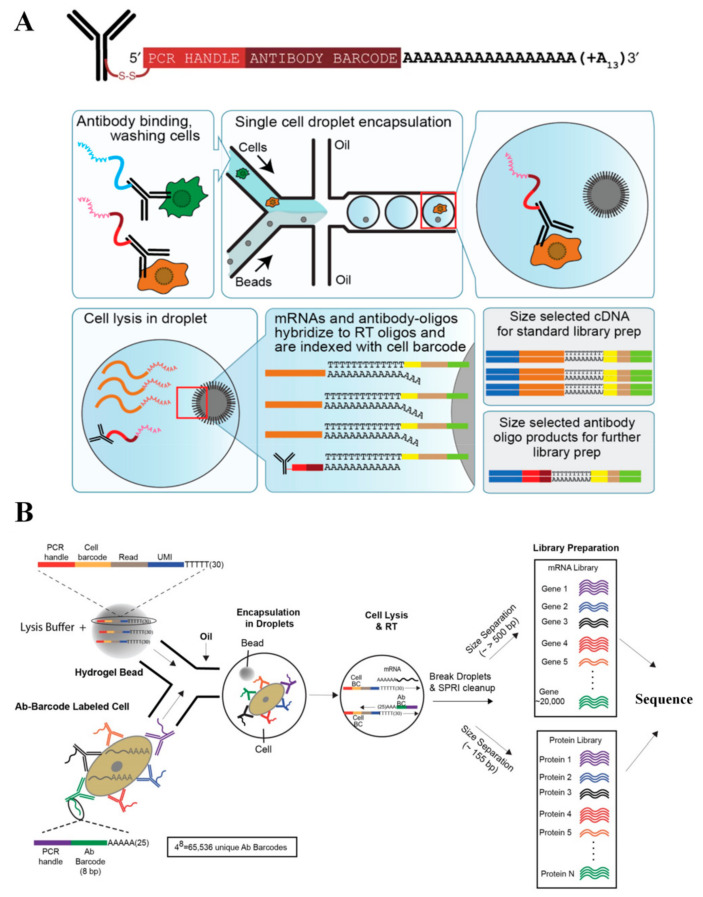
Combination of scRNA-seq with proteomic analyses. (**A**) Cite-seq is introduced to analyze cell transcriptomes alongside surface protein abundance on the single cell level. Adapted with permission from Reference [62] © Creative Commons Attribution License (2020). (**B**) Reap-seq enables simultaneous quantification of 82 proteins and mRNAs from single cells. Adapted with permission from Reference [60] © Springer Nature (2017). Both methods have shown a more detailed characterization of the cellular phenotype than transcriptome measurements alone.

Cellular indexing of transcriptomes and epitopes by sequencing (Cite-seq) was introduced to simultaneously analyze transcriptomes alongside cell surface protein abundance at the single cell level [59] (Figure 6A). The method involves the linkage of the 5′ end of oligos to antibodies through streptavidin-biotin interactions. The antibodies are streptavidin labeled and the DNA oligonucleotides with a 5′ amine modification are biotinylated using NHS-chemistry. This process results in the formation of a disulphide bond that separates the oligonucleotide from the antibody in reducing conditions. By adding 50 mM dithiothreitol (DTT) buffer, it is possible to cleave the disulfide bond in the spacer arm of the biotin attached to the oligonucleotides, which produces DNAs with high purity. Single cells are sorted and lysed. The mRNA and antibody-tagged oligonucleotides are bound to oligo(dT) primers on magnetic beads. The antibody-tagged oligonucleotides contain a barcode for identification along with a handle for PCR amplification. ScRNA-seq was performed following the Drop-seq protocol. The proposed method allows simultaneous detection of about 13 surface proteins and transcripts.

### 4.2. Reap-seq

Similar to Cite-seq, RNA expression and protein sequencing (Reap-seq) enables parallel quantification of mRNAs and protein at a single cell resolution [60] (Figure 6B). It enables the detection of about 82 antibodies and over 20,000 genes. The two approaches differ in how the DNA barcode is conjugated to the antibody. Unlike Cite-seq, Reap-seq utilizes unidirectional chemistry that generates a stable and small covalent bond between the aminated DNA barcodes and antibody, which reduces steric hindrance and potential crosstalk. Minimizing steric hindrance is essential in high-throughput protein analysis and in the future extension of this method to intracellular labeling. This approach is readily adaptable to the existing scRNA-seq platforms, which shows a more detailed assessment of both the transcriptome and cellular phenotype than transcriptome measurements alone.

Both Cite-seq and Reap-seq used a similar approach, generating a protein readout to be sequenced alongside a single cell transcriptome. Unlike the detection of cell surface protein by flow cytometry, these technologies use DNA barcodes to label surface antibodies. DNA barcoding enables the combination of various antibodies by targeting distinct epitopes in a single assay that could be resolved through sequencing. This study has enabled a more comprehensive analysis of single cells, which allows fine discrimination between cell types. This was unobtainable with mRNA data alone. It could also be potentially used in exploring post-transcriptional gene regulation. To date, extending the technology to detect intracellular proteins alongside transcriptomes remains a challenge, particularly due to the requirement of cell permeabilization, which may cause RNA degradation.

### 4.3. PDMS Nanowell and seq

To detect both transcriptome and secretome from the same cells, one study developed a PDMS-Nanowell technology to study cytokine secretion, which is followed by downstream transcriptomic analysis [61]. The PDMS is collagen-coated and loaded with single cells. The entire device is subsequently sealed with an antibody array, which captures cytokines secreted by single cells. Single cells with a desirable secretion profile (i.e., high tumor necrosis factor (TNF)-α secretors) are picked up using a 32G syringe for subsequent RNA sequencing. Through transcriptomic analysis, a subgroup of highly co-expressed genes correlating with TNF-α secretion in mouse macrophage cells was discovered. This technology may lead to a deeper understanding of the immune regulatory mechanism, which shows a great promise for drug discovery and medical therapy.

## 5. Combination of scRNA-seq with DNA Analysis

The existing scRNA-seq approaches do not provide genotypic information such as gDNA copy number, chromosome structure, and number. In fact, paired transcriptomic and DNA analysis reveals the effects of changes in the DNA structure and copy number on gene expression. This would directly link the cell genotype (wild-type or mutant) to its functional states (cell types and states) [34]. For instance, the cancer driving genes that directly affect downstream gene expression and regulate metastasis could be identified through both transcriptomic and DNA analyses [63]. This integrative analysis would enhance our understanding of population architectures and cellular properties of heterogeneous healthy and diseased tissues. Current studies analyze transcriptomic and genomic data separately. For example, in one study, both NODES algorithm and the Genome Analysis Toolkit variant calling pipeline were used to analyze scRNA-seq and scDNA-seq data, respectively [64].

To integrate DNA analysis and the transcriptome measurement into an efficient, single cell readout, several technologies have been developed, which are described below. These technologies are summarized in Table 4.

### 5.1. DR-seq

**Figure 7:**
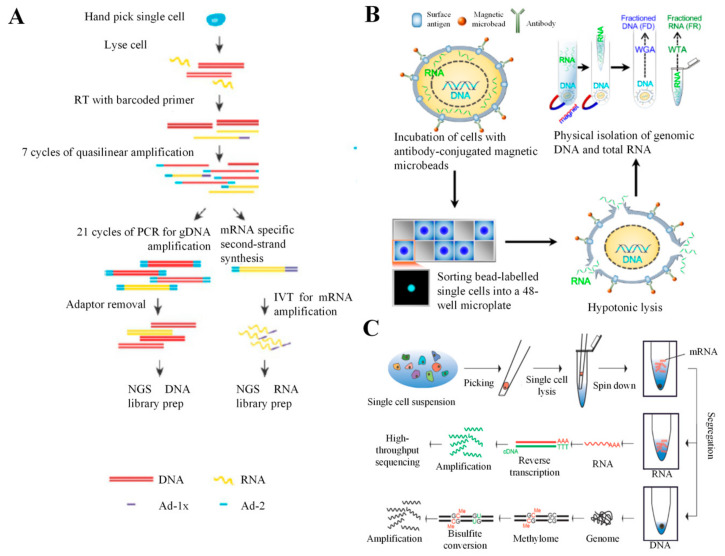
Combination of scRNA-seq with DNA analysis. (**A**) DR-seq permits simultaneous transcriptomic and DNA analysis of the same single cell using a quasilinear amplification strategy. Adapted with permission from Reference [65] Springer Nature (2015). (**B**) SIDR-seq physically separates all RNAs from gDNA, allowing polyadenylated and non-polyadenylated RNAs to be collected for scRNA-seq. Adapted with permission from Reference [67] © Creative Commons Attribution License (2017). (**C**) ScTrio-seq performs genome and transcriptome sequencing as well as DNA methylome analysis simultaneously. Adapted with permission from Reference [68] © Creative Commons Attribution License (2016).

gDNA-mRNA sequencing (DR-Seq) permits simultaneous transcriptomic and DNA analysis of the same single cell using a quasilinear amplification strategy without physically separating mRNA from gDNA (Figure 7A) [65]. A single cell is picked using a mouth pipette and deposited into a PCR tube. The cell is lysed to release RNA and gDNA. The mRNA is reverse transcribed to single stranded cDNA using adaptor 1-x (Ad-1x) and a poly-T primer having a cell-specific barcode (5′ Illumina adaptor and a T7 promoter overhang). Then, both the cDNA and gDNA are subjected to quasilinear whole-genome amplification (WGA) using adaptor-2, which is known to have a defined 27-nt sequence at the 5′ end, which is followed by eight random nucleotides. Following amplification processes, most amplicons have AD-2 at both ends. A small number of cDNA-derived amplicons with Ad-2 at one end and Ad-1x at the other end are generated. Half of the sample is subjected to PCR, which is followed by Ad-2 removal and preparation of a cell-specific indexed Illumina library for sequencing of gDNA. The other half is converted to double-stranded cDNA, which is followed by amplification using in vitro transcription and preparation of NGS RNA library for transcriptomic analysis. DR-Seq was found to perform efficiently as the existing scRNA-seq methods, including CEL-seq and MALBAC. Additionally, DR-seq results revealed that variability of gene expression between single cancer cells could be contributed by gDNA copy-number variation [65]. Therefore, DR-seq could be applied to determine transcriptional consequences of the gDNA copy number variations in diseased and healthy tissues. Since mRNA and DNA are amplified without physical separation, DR-seq requires in silico masking of coding sequences during analysis to determine gDNA copy-number variation.

### 5.2. G and T-seq

Similar to DR-seq, genome and transcriptome sequencing (G and T-seq) enables simultaneous single cell DNA and transcriptomic analysis to evaluate the effects of genetic variation on gene expression [66,69]. A single cell can be isolated either via FACS or manually by a micro-pipettor and deposited into a well of a 96-well plate. The cell is lysed to release RNA and gDNA, which is followed by physical separation of mRNA from gDNA using biotinylated oligo(dT) primer functionalized on streptavidin magnetic beads. After mRNA is bound with the beads, a magnet is placed under the plate to capture the beads, allowing the collection of supernatant containing gDNA. The mRNA is subjected to on-bead WTA prior to transcriptome sequencing, whereas the gDNA is subjected to WGA prior to genome sequencing. G and T-seq was found to be capable of dictating the transcriptional consequences of chromosomal abnormalities (e.g., inter-chromosomal fusions and chromosomal aneuploidies) in a single cell. Therefore, G and T-seq could help establish the functions of cell-to-cell variation in the chromosomal structure and number in disease and normal development processes. In contrast to DR-seq, G and T-seq does not require in silico masking of coding sequences for identifying genomic copy-number variation that simplifies the data analysis processes.

### 5.3. SIDR-seq

In addition to the above-mentioned technologies, simultaneous isolation and parallel sequencing of gDNA and total RNA (SIDR-seq) was also introduced to simultaneous sequence gDNA and RNA (Figure 7B) [67]. Bulk cells are first bound to the cell-specific antibody-conjugated magnetic microbeads, which is followed by sorting of bead-labelled single cell into a well of a 48-well microplate. A hypotonic solution is used to disrupt the plasma membrane of a single cell to release all cytoplasmic RNA while gDNA remained within the nucleus. A magnet is then placed under the plate to capture the bead-labelled single cell to physically separate RNA from gDNA, allowing collection of supernatants containing total RNA. The mRNA is reverse-transcribed and subjected to WTA prior to transcriptome sequencing, whereas the gDNA is subjected to WGA prior to genome sequencing. SIDR-seq offers some advantages over DR-seq and G and T-seq [67]. First, SIDR-seq does not require in silico masking of coding sequences during analysis for identifying genetic variants. Second, SIDR-seq can physically separates all RNAs from gDNA, including mRNA and non-coding RNAs (particularly long noncoding RNA), which allows long noncoding RNA to be collected for potential application in a cancer diagnosis [70]. In addition, SIDR-seq demonstrated higher rates of alignment than DR-seq or nuc-seq (single cell genome sequencing method) and lower rates of duplication than DR-seq. This technology could be applied for a more comprehensive study of cellular heterogeneity and complexity at a single-cell resolution.

### 5.4. CORTAD-seq

Concurrent sequencing of the transcriptome and targeted genomic regions (CORTAD-seq) allows simultaneous evaluation of the transcriptome and genome within the same single cell in an automated, high-throughput microfluidic platform (Fluidigm C1) [64]. The Fluidigm C1 can capture and process up to 96 single cells for gDNA and mRNA sequencing. Single cells are lysed to release RNA and gDNA, which is followed by conversion of mRNA to cDNA. Both gDNA and cDNA are then subjected to PCR using primers specific to the regions of interest. Half of the sample is subjected to another round of PCR using genotyping primers for amplifying gDNA while reducing the amount of cDNA prior to genome sequencing. The other half is directly used for transcriptome sequencing. CORTAD-seq revealed that the transcriptome of the lung cancer cell undergoing a T790M mutation is slightly different from that of a T790M wild-type lung cancer cell. Therefore, it could be applied to study the transcriptomic consequences of the known targeted gene mutations in various types of cancer. With the requirement of having known targeted sequences, this technology is not suitable for genome-wide DNA analysis for a discovery purpose.

### 5.5. scTrio-seq

Besides genome and transcriptome sequencing, single-cell triple omics sequencing (scTrio-seq) also analyzes epigenome or DNA methylome from the same cell (Figure 7C) [68]. It has been reported that epigenomics plays important roles in regulating gene expression of a single cell [71]. A single cell is first transferred into a PCR tube using a mouth pipette and lysed to release only mRNA. Following centrifugation, mRNA is physically separated from the intact nucleus containing DNA, which allows the collection of supernatants containing mRNA for RT and cDNA amplification (Smart-seq or CEL-seq) prior to transcriptome sequencing. The intact nucleus is lysed, and the released gDNA is subjected to WGA and bisulfite-converted for genome sequencing and DNA methylome sequencing, respectively. scTrio-seq results demonstrated that DNA methylation at the promoter downregulates gene expression in a single cell, and DNA methylation at a gene body upregulates gene expression in a single cell. Based on the multi-omics information of each single cancer cell, different subpopulations of cancer cells can be identified, and the malignancy and metastasis potential of the subpopulations could be determined [68]. Taken together, scTrio-seq could be applied to determine transcriptional consequences of genomic and epigenomic heterogeneities within a population of cells especially cancer cells.

## 6. Commercial scRNA-seq Technologies

**Figure 8:**
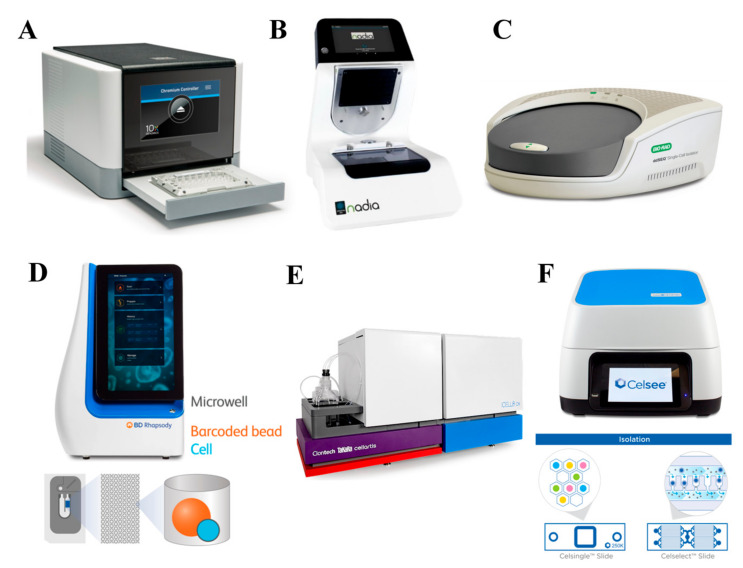
Commercial scRNA-seq technologies. There are several scRNA-seq technologies available in the market such as the (**A**) chromium system (10x genomics) (adapted with permission from Reference [54] © Creative Commons Attribution License 2017), (**B**) Nadia (Dolomite Bio) (adapted with permission from Reference [72] © Creative Commons Attribution License 2018), (**C**) ddSEQ single cell isolator (Illumina, Bio-Rad) (adapted with permission from Reference [2] © Springer Nature 2018), (**D**) BD Rhapsody single cell analysis system (BD) (adapted with permission from Reference [73] © Springer Nature 2019), (**E**) ICell8 single cell system (Takara) (adapted with permission from Reference [74] © Creative Commons Attribution License 2017) and (**F**) Celselect Technology (Celsee) (adapted with permission from Reference [72] Creative Commons Attribution License 2018).

To date, there are a few commercial scRNA-seq sample preparation technologies, including droplet-based and Nanowell-based technologies (Table 1). Droplet-based technologies such as chromium system (10x genomics) (Figure 8A) [54] and in-Drop system (1CellBio) [24] have enabled high-throughput single cell analysis (>10,000 cells) with intensive user support. Similarly, Nadia (Dolomite Bio) was introduced for scRNA-seq (Figure 8B) [72]. It is a fully automated technology using the principle described in the DropSeq protocol. The other droplet-based technology, namely ddSEQ single cell isolator (Illumina, Bio-Rad), was also developed to use microfluidic cartridges to encapsulate cells and barcodes into droplets (Figure 8C) [2]. The library preparation methods are similar to that of other droplet-based technologies. While these technologies are advanced, they possess some limitations such as droplet fragility, risk of leakage, and poor cell capturing efficiency especially when the initial cell density is low. High initial cell concentration would, otherwise, increase the chance of having doublets in each droplet or of clogging the system.

To address these drawbacks, some producers utilize Nanowell-based technologies such as a BD Rhapsody single cell analysis system (BD) (Figure 8D) [73], ICell8 single cell system (Takara) (Figure 8E) [74], C1 System and Polaris (Fluidigm) [75], and Celselect Technology (Celsee) (Figure 8F) [72]. These technologies have more than thousands of Nanowells with each consisting of a single bead conjugated with oligonucleotides to capture target mRNAs, which enables high-throughput analysis. Before cell lysis, the array is observed and the information such as bead numbers, cell numbers, numbers of cell doublets, and empty wells are recorded. The number of beads and cells can be optimized to obtain maximum sequencing efficiency. Unlike droplet-based technologies, they are user-friendly and readily operated by untrained users. They are also able to process small numbers of cells such as rare cells. In addition, most of them can perform cell selection based on cell characteristics, surface markers, or morphology.

To easily isolate single cells of interest for downstream processes, the puncher platform (Vycap) was introduced [76]. The main advantage of this technology is the ability to isolate rare single cells (e.g., circulating tumor cells) from real samples [77,78]. The sample is filtered through the cell isolation chip, which consists of more than 6000 Nanowells with a single micropore. To allow the cell suspension to flow through the micropores, low pressure is applied to sort the single cells into individual wells in a few minutes. The bottom of each well containing a single cell is then punched out using a punch needle and collected into a microplate or a tube. This technology has successfully collected more than 95% of selected cells, which could be potentially used for various scRNA-seq applications.

## 7. Conclusions

In summary, recent development of scRNA-seq technologies such as valve-based, droplet-based, and Nanowell-based scRNA-seq technologies have enabled highly-sensitive, accurate, and high throughput transcriptomic analysis of individual cells. Combining single-cell transcriptomic data with proteomic data enables understanding of how transcriptomic cellular states translate into functional phenotypic states. In addition, it may reveal phenotypic cell states not obtainable from scRNA-seq data alone due to the fact that heterogeneity may present in both post-transcriptional and post-translational processes. Integrating transcriptomic data with genomic data allows detection of a gene mutation alongside transcriptomes. This integration could gain insights into cancer evolution and help address medical challenges not obtainable from RNA sequencing alone, such as dissecting complex cellular immune responses or determining intra-tumor heterogeneity.

One area of future research will be improving efficiency of current technologies including sensitivity, multiplexing, throughput, and cost-effectiveness. Specifically, given the requirement of analyzing more cells, the cost of consumables, labor, and sequencing remains high, which poses a barrier for widespread implementation of most scRNA-seq technologies. Therefore, reducing total cost might be a crucial step. Developing high-throughput technologies (e.g., droplets or Nanowell-based technologies) with a high-sample processing capacity and low reaction volume along with easily fabricated and operated processes could achieve efficient scRNA-seq at a minimal cost. The automation of devices would reduce assay time and minimize human intervention [64]. This will limit user bias and improve reproducibility across different laboratories. While achieving the above-mentioned criteria, assay sensitivity should not be compromised. In addition, current technologies rely on polyT priming, and, hence, only polyadenylated mRNAs are sequenced, which may not be informative [23]. Sequencing of non-polyadenylated RNA such as long non-coding RNA is important to gain a better understanding of gene regulation and gene expression heterogeneity. Therefore, it would be helpful to develop technologies that allow sequencing of these molecules to improve efficiency and quality of scRNA-seq.

Future progress could explore multi-omics to gain a more comprehensive outlook of cell types and cell states. For example, DNA sequencing and DNA methylation could be detected alongside transcriptomes using long-read sequencing to obtain more cellular information [68]. Meanwhile, specialist multi-omics algorithms should be created to analyze different layers of data simultaneously. High resolution capture of spatial information integrated with scRNA-seq should be extended to include antibody tags or other nucleic acid-tag [59]. In addition, a greater linkage of live-cell imaging data with scRNAseq data may allow the interrogation of more complex phenotypes. For instance, cell imaging prior to sequencing could provide information about cellular phenotypes (e.g., cell size, morphology, and surface markers) and dynamics (e.g., cell growth proliferation, differentiation, migration, and cell-cell interaction), which can then be correlated with the transcriptome of the same cells by providing a more informative output. Incorporating 3D hydrogels such as polyethylene glycol diacrylate or gelatin methacryloyl into technologies could lead to a better understanding of an in vivo single cell response [79,80]. In addition, the use of combinatorial indexing or an optically decodable bead [57] could link live cell microscopy assays to scRNA-seq as well as to detect multiplets. More importantly, a precise and facile cell retrieval technology for downstream analysis is desirable.

Besides technological improvements, computational analytical methods are also improving. The sources of review articles on scRNA-seq bioinformatics are extensive and readily available [35,81,82]. One of the challenges in analyzing scRNA-seq data is the presence of technical noise due to low initial cell concentration or poor efficiency of transcript detection, which results in variability in sequencing efficiencies in different cells [35]. The noise source can be modeled using spike-in RNA standards. It is challenging to identify the repertoire of cell subpopulations and various genes between them. Therefore, it is recommended to select a subset of known transcription factors or genes relevant to the biological information of the specific tissue. It is also suggested to use clustering algorithms (e.g., hierarchical clustering, K-means clustering) [83] to identify specific cell types based on the expression of these genes across various cells. Additionally, future work should include the generation of two or more orders of magnitude larger data to produce more single-cell information in a short period of time. We anticipate that, in the future, more studies focusing on developing robust scRNA-seq technologies will help unravel the function of individual cells in a variety of human diseases and show tremendous promise for biological and clinical applications.

## Figures and Tables

**Table 1:** Summary of conventional scRNA-seq technologies.

Technology	Cell Isolation Method	No. of Cells	Cell Barcode	Unique Molecular Identifiers	cDNA Coverage	Amplification Method	Advantages	Limitations	Outcomes
Smart-seq 1 & 2 [37,38]	Micropipette	100–1000	No	No	Full-length	Template switching-based PCR	Increased throughput and read coverage across transcripts Smart-seq 2 increases thermal stability of LNA-DNA base pairs.	Low number of cells Time-consuming cell isolation processes	Transcript enumeration Analysis of alternative splicing allelic expression Investigation of transcriptomic profile in rare cells
CEL-seq 1 and 2 [13,14]	Micropipette	100–1000	Yes	Yes	3′ tag	In vitro transcription-based 3′ transcript amplification * The protocol is based on Smart-seq	CEL-Seq 2 adds a 5-base pair UMI upstream of the barcode to distinguish between PCR duplicates and transcript abundance in scRNA-seq, which significantly improves accuracy.	3′ end sequencing only The use of micropipette for cell isolation makes the operational processes more difficult and time-consuming. Low number of cells are processed.	It is used to study early *C. elegans* embryonic development at single cell level. CEL-seq will be useful for transcriptomic analyses of complex tissues containing populations of diverse cell types.
SCRB-seq [15]	FACS	1000–10,000	Yes	Yes	3′ tag	Template switching-based PCR * The protocol is based on Smart-seq.	High throughput	Requires skilled workers	Characterization of primary human adipose-derived stem cell differentiation system Discovery of transcriptomes across heterogeneous populations
MARS-seq 1 & 2 [39,40]	FACS	1000–5000	Yes	Yes	3′ tag	In vitro transcription-based 3′ transcript amplification	Automated processes minimize amplification bias and labeling errors	Requires skilled workers	Analysis of in vivo transcriptional states in thousands of single cells. Identification of a unique microglia type that may restrict the development of Alzheimer’s disease
Quartz-seq 1 [41]	FACS	1000–10,000	No	No	Full length with 3′ biased	PCR after poly(A) tailing	Highly quantitative	Requires skilled workers	Detection of transcriptome heterogeneity between the cells in the same and different cell-cycle phases
Quartz-seq 2 [42]	FACS	1000–10,000	Yes	Yes	Full length with 3′ biased	PCR after poly(A) tailing	Able to detect more transcripts from limited sequence reads at a minimal cost	Requires skilled workers	Detection of transcriptome heterogeneity between embryonic stem cells and between cells in stromal vascular fraction
SUPeR-seq [43]	Mouth pipette	~10	Yes	No	Full length	PCR after poly(A) tailing	Able to detect both circular RNA (non-polyadenylated RNA) and polyadenylated RNA	Low throughput Operational processes are difficult and time-consuming	Analysis of expression dynamics of circular RNA during mammalian early embryonic development
MATQ-seq [44]	Mouth pipette	10–100	Yes	Yes	Full length	PCR after poly(A) tailing	Able to sequence both polyadenylated and non-polyadenylated RNAs with high sensitivity and accuracy	Low throughput Operational processes are difficult and time-consuming	Detection of low abundance genes and non-polyadenylated RNA extracted from a single cell

**Table 2:** Summary of microfluidic-based scRNA-seq technologies.

Technology	Cell Isolation Method	No. of Cells	Cell Barcode	Unique Molecular Identifiers	cDNA Coverage	Amplification Method	Advantages	Limitations	Outcomes
Multilayer microfluidic device and seq [19]	Valve	10–100	Yes	No	Full-length	PCR after poly(A) tailing	Improvement of assay sensitivity The semi-automated processes minimize technical variation and reduce risk of contamination	The requirement of off-chip amplification Complex device fabrication processes Low throughput	Identification of differentially expressed genes of single cells and measurement of biological variations in cell populations.
Microfluidic hydrodynamic trap array & seq [50]	Valve	10–5000	Yes	No	Full-length	Template switching-based PCR * The protocol is based on Smart-seq 2	Allows multi-generational lineage tracking under controlled culture conditions	Complex device fabrication processes	Measurement of the effects of lineage and cell cycle-dependent transcriptional profiles of single cells.
MID-RNA-seq [51]	Valve	1000	Yes	No	Full length	PCR after poly(A) tailing	Allows automated processing and multiplexing	Complex device fabrication processes	Transcriptomic studies of scarce cell samples.
Hydro-seq [52]	Valve	10–1000	Yes	Yes	3′ tag	Template switching-based PCR * The protocol is based on Drop-seq.	Improved throughput and cell capture efficiency	Complex device fabrication processes	Identification of cellular heterogeneity in critical biomarkers of tumor metastasis, understanding tumor metastasis processes, and monitoring target therapeutics in cancer patients.
Hi-SCL [53]	Droplet	1000–10,000	Yes	No	3′ tag	PCR after poly(A) tailing	High throughput	Low cell capture efficiency	Detection and comparison of transcriptomes in mouse embryonic stem cells and mouse embryonic fibroblast populations at the single-cell level.
In-drop [24]	Droplet	1000–10,000	Yes	Yes	3′ tag	In vitro transcription-based 3′ transcript amplification * The protocol is based on CEL-seq.	High throughput	Low cell capture efficiency Only the 3′ most terminal fragments can be used for sequencing	Sequencing of large numbers of cells from heterogeneous populations in a fast way and identification of very rare cell types.
Drop-seq [23]	Droplet	1000–10,000	Yes	Yes	3′ tag	Template switching-based PCR	High throughput, cheaper, and faster	Only the 3′ most terminal fragments can be used for sequencing	Analysis of mRNA transcripts from thousands of individual cells concurrently while identifying the cell of origin.
10x Genomics [54]	Droplet	1000–10,000	Yes	Yes	3′ tag	Template switching-based PCR	The use of 10x barcodes significantly increase throughput	Only the 3′ most terminal fragments can be used for sequencing	Profile 68k peripheral blood mononuclear cells and dissect large immune populations.
MULTI-seq [55]	Droplet	10,000–100,000	Barcoded lipid-modified oligonucleotides	Yes	3′ tag	Template switching-based PCR * The protocol is based on 10x genomics.	Readily multiplex various cell types and identify cell doublets	Only the 3′ most terminal fragments can be used for sequencing	Assessment of immune cell responses to tumor metastatic progression.
Cytoseq [56]	Nanowell	100–10,000	Yes	Yes	3′ tag	Gene specific primers-based PCR	High throughput Simple fabrication and operation processes	Not fully automated	Characterization of cellular heterogeneity in immune response and identification of rare cells in a cell population.
Microwell-seq [26]	Nanowell	100–10,000	Yes	No	Full-length	Template switching-based PCR * The protocol is based on Smart-seq 2.	High throughput Simple fabrication and operation processes	Not fully automated	Construction of “mouse cell atlas” with more than 400k single-cell transcriptomic profiles from 51 mouse tissues, organs, and cell cultures, covering more than 800 major cell types and 1000 cell subtypes in the mouse system.
Seq-well [25]	Nanowell	100–10,000	Yes	Yes	3′ tag	Template switching-based PCR * The protocol is based on Drop-seq.	High throughput Simple fabrication and operation processes The use of semipermeable polycarbonate membrane reduces well-to-well contamination	Not fully automated	Profile thousands of primary human macrophages exposed to *Mycobacterium tuberculosis*. It is compatible with on-array imaging cytometry for resolving the phenotype of cells from complex samples.
SCOPE-seq [57]	Nanowell	100–10,000	Yes	Yes	3′ tag	Template switching-based PCR * The protocol is based on Drop-seq.	High throughput Simple fabrication and operation processes The phenotypes measured can be directly linked to expression profiles using optically decodable beads The use of perfluorinated oil prevents well-to-well contamination	Not fully automated	Combination of live cell imaging with single-cell RNA sequencing for various biomedical applications.
scFTD-seq [58]	Nanowell	100–10,000	Yes	Yes	3′ tag	Template switching-based PCR * The protocol is based on drop-seq.	High throughput Simple fabrication and operation processes Minimizing contamination by preventing immediate cell lysis	Not fully automated	Profile circulating follicular helper T cells implicated in systemic lupus erythematosus pathogenesis

**Table 3:** Summary of integration of scRNA-seq with protein analysis.

Technology	Cell Isolation Method	No. of Cells	Cell Barcode	Unique Molecular Identifiers	cDNA Coverage	cDNA Amplification Method	Advantages	Limitations	Outcomes
Cite-seq [59]	Droplet	1000–10,000	Yes	No	3′ tag	Template switching-based PCR *The protocol is based on Drop-seq.	High throughput Allows simultaneous transcriptomic and surface protein analysis	Low cell capture efficiency Not fully automated	Simultaneous detection of about 13 surface proteins and transcripts
Reap-seq [60]	Droplet	1000–10,000	Yes	Yes	3′ tag	Template switching-based PCR * The protocol is based on 10x genomics.	High throughput Allows simultaneous transcriptomic and surface protein analysis	Low cell capture efficiency Not fully automated	Assessment of costimulatory effects of a CD27 agonist on human CD8+ lymphocytes and characterization of an unknown cell type
PDMS Nanowells and seq [61]	Nanowell	1000–10,000	Yes	No	Full length	Template switching-based PCR * The protocol is based on Smart-seq 2.	High throughput Allows simultaneous transcriptomic and secretion analysis	Not fully automated	Study of regulation mechanisms of the immune system

**Table 4:** Summary of integration of scRNA-seq with DNA analysis.

Technology	Cell Isolation Method	No. of Cells	Cell Barcode	Unique Molecular Identifiers	cDNA Coverage	cDNA Amplification Method	Advantages	Limitations	Outcomes
DR-seq [65]	Mouth pipette	10–50	Yes	No	3′ tag	In vitro transcription * The protocol is based on CEL-seq.	Allows simultaneous transcriptomic and DNA analysis	Complex work flow and low throughput Requires in silico masking of coding sequences, which complicates the data analysis processes	Study of transcriptional consequences of gDNA copy number variations in diseased and healthy tissues
G and T-seq [66]	FACS	10–100	No	No	Nearly full length	Template switching-based PCR * The protocol is based on Smart-seq 2.	Simple work flow Allows simultaneous transcriptomic and DNA analysis	Requires skilled workers Low throughput	Study of transcriptional consequences of chromosomal abnormalities in a single cell
SIDR-seq [67]	Micropipette	10–100	No	No	Nearly full-length	Template switching-based PCR * The protocol is based on Smart-seq 2.	Automated and simple work flow Allows simultaneous transcriptomic and DNA analysis	Low throughput	Assessment of cellular heterogeneity in breast and lung cancer at the singular cell level
CORTAD-seq [64]	Fludigm C1	100–1000	No	No	Full length with weak 3′-biased	Template switching-based PCR * The protocol is based on Smart-seq.	Automated and high throughput Allows simultaneous transcriptomic and DNA analysis	Not suitable for genome-wide DNA analysis for discovery purpose	Study of transcriptional consequences of known targeted gene mutations in various types of cancer
scTrio-seq [68]	Mouth pipette	10–50	No	No	Full length with weak 3′-biased	Template switching-based PCR * The protocol is based on Smart-seq.	Allows simultaneous transcriptomic, genomic, and epigenomic analysis	Complex work flow and low throughput	Study of transcriptional consequences of genomic and epigenomic heterogeneities within a population of cells especially cancer cells
